# Changes in medical students’ motivation and self-regulated learning: a preliminary study

**DOI:** 10.5116/ijme.565e.0f87

**Published:** 2015-12-28

**Authors:** Kyong-Jee Kim, Hye W. Jang

**Affiliations:** 1 Department of Medical Education, School of Medicine, Dongguk University, South Korea; 2 Department of Medical Education, School of Medicine, Sungkyunkwan University, South Korea

**Keywords:** Medical students, motivation, self-regulated learning, Korea

## Abstract

**Objectives:**

To investigate whether medical students’ motivation and Self-Regulated Learning (SRL) change over time to enhance our understanding of these constructs as dependent variables in medical education.

**Methods:**

A cohort of first-year students (n=43) at a medical school in South Korea completed a self-report questionnaire on motivation and SRL - the Motivated Strategies for Learning Questionnaire (MSLQ). The same questionnaire was administered to the same cohort in the beginning of Year 2. A Wilcoxon signed-rank test was conducted to determine if changes in participants’ MSLQ scores occurred between in Years 1 and 2.

**Results:**

Forty-one students completed the questionnaires in both years (95% response rate). Participants’ motivation scores significantly increased, whereas their SRL scores decreased significantly after they went through Year 1. The most notable change in participants’ MLSQ scores was in the increase in their test anxiety. There was a positive association between the participants’ test anxiety and their cognitive strategies use in Year 1, which changed to a negative one in Year 2. Meanwhile, participants’ test anxiety scores and their self-regulation scores became more negatively associated over time.

**Conclusions:**

Our study shows that even as medical students become more motivated, they actually use fewer self-regulated strategies over time. Our findings highlight the need for change in the medical school’s learning environment to lessen students’ test anxiety to facilitate their use of cognitive and meta-cognitive strategies.

## Introduction

Theories and research suggest that students’ motivation and self-regulated learning, which can be conceptualized as students participating meta-cognitively, motivationally, and behaviorally actively in their learning,^1^ are linked to their cognitive engagement and academic achievement.^2^ Accordingly, motivation and self-regulated learning have garnered attention in medical education research. Research has also related student motivation and self-regulated learning to his or her performance in medical school.^3^ Consequently, medical school curricula are increasingly called upon to promote students’ self-regulated learning.^4^

Theories and research suggest that motivation and self-regulated learning are not only mediate learning but are also consequences of the learning; thus, they are both dependent and independent variables in medical education.^1^^,^^5^^,^^6^ Furthermore, an individual’s motivation and self-regulated learning may differ according to his or her backgrounds, such as age and gender.^6^^-^^8^ Therefore, it can be hypothesized that students’ motivation and self-regulated learning change over time in medical school and these changes are affected by several factors. Still, research on motivation and self-regulated learning as dependent variables to student learning and performance is scant in the field of medical education and therefore empirical evidence is lacking.

The purpose of this study was to investigate changes in medical students’ motivation and self-regulated learning over time in their medical studies. In doing so, this study was meant to enhance our understanding of motivation and self-regulated learning as dependent variables in medical education, which has implications for inventions for promoting medical students’ motivation and self-regulated learning.

## Methods

The study participants were medical students at Sungkyunkwan University School of Medicine (SKKUSOM) in South Korea.  Approximately forty students were admitted to SKKUSOM annually at the time of this study: half graduate entrants and half undergraduate entrants. The curriculum is a four-year program composed of mostly lecture-based courses in basic medical sciences in Year 1, and pre-clinical courses in the problem-based learning format in Year 2, followed by two-year clinical clerkships. Undergraduate-entry students undertake two-year premed courses prior to this four-year curriculum.

We measured participants’ motivation and self-regulated learning using five sub-scales adapted from the Motivated Strategies for Learning Questionnaire (MSLQ). Based upon the social cognitive theory of learning, MSLQ is a self-report instrument designed to assess students’ motivational orientations and their use of different learning strategies.^2^ This instrument is applicable to and has been studied in various educational settings - from elementary classrooms to college courses - and is known to have a reasonable predictive validity in students’ academic achievement.^2^ A few studies were conducted in medical education research using MSLQ and they also found association between medical students and trainees’ MSLQ scores and their academic performance.^3^^, ^^9^^-^^11^

The questionnaire consists of 44 items encompassing motivation and self-regulated learning components. The motivation component includes three sub-scales: self-efficacy (9 items), intrinsic value (9 items), and test anxiety (4 items). The self-regulated learning component includes two sub-scales: cognitive strategy use (13 items: e.g., use of rehearsal, elaboration, and organizational strategies) and self-regulation (9 items related to use of metacognitive strategies - e.g., planning, skimming, and comprehension monitoring - and effort management). The items were rated on a five-point Likert scale, from 1 being “strongly disagree” to 5 being “strongly agree”. The original version in the English language was translated into Korean by the investigators and was reviewed by experts for validation. The questionnaire was also pilot tested several times in the previous years by the authors. Reliability analysis was performed to examine the internal consistency of this instrument. The Cronbach’s alpha ranged from 0.83 to 0.90, which is considered an acceptable range.^12^

The same questionnaire was administered twice to the same cohort of students across two academic years to investigate changes in their MSLQ scores over time in the medical program. The first survey was conducted in the first week of Year 1 in March, 2013. The second survey was administered a year later in the first week of their second year in medical school, February, 2014.

Institutional review board (IRB) approval was not requested for the present study, because it was part of the annual survey of new incoming students, which fell under the general exemption from our IRB for educational outcomes data.

The data were subjected to descriptive analysis in order to calculate means for each sub-scale in MSLQ. Additionally, A Wilcoxon signed-rank test was conducted to determine if changes in participants’ MSLQ scores occurred between in Years 1 and 2. Furthermore, participants’ Year 1 GPAs were compared across genders and across different entry levels using a Mann-Whitney U test to compare their baseline performance. Correlation anslysis was also performed to investigate relationpships between variables. We used SPSS version 20 for Windows for all statistical analyses. All significance was tested at the 95% level of confidence.

## Results

All first-year students (n=43) were invited and agreed to participate in this study. Two of the participants who completed the questionnaire in Year 1 were excluded from the second survey as they had failed to progress and therefore were dropped out of the cohort. Consequently, 41 participants (95.3% of the original cohort) completed the questionnaires in both years, of whom 70% (n=29) were male, 30% (n =12) were female. 46% (n=19) of the participants were undergraduate-entry and 54% (n=22) were graduate-entry students. Participant ages ranged from 20 to 29 (M= 22.8 ± 1.93).

**Figure 1:**
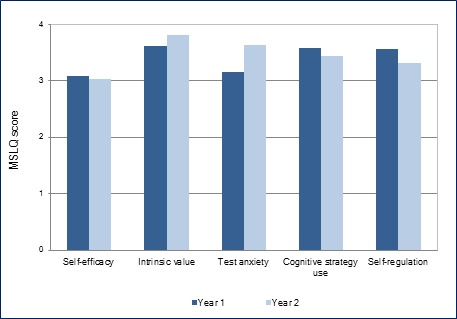
Changes in participants’ MSLQ scores between the two years (n = 41)

Participants’ Year 1 GPAs differed neither between genders (Z=1.94, p =.11) nor between undergraduate-entry and graduate-entry students (Z=.98, p=.63). There were no significant differences in participants’ MSLQ scores in Year 1 across genders, where Z ranges from .71 to .10 and p ranges from .92 to .47, or across groups of different entry levels, where Z ranges from 1.33 to .32 and p ranges from .85 to .07.  Figure 1 shows changes in participants’ MSLQ scores between Year 1 and 2. In terms of the motivational component in MSLQ, participants’ intrinsic value and test anxiety scores increased significantly (Z =2.07, 3.15, respectively, p < .05), whereas there were no significant changes in their self-efficacy scores (Z=1.01, p=.31).

Meanwhile, participants’ MSLQ scores in both sub-scales in the self-regulated learning component (i.e., cognitive strategy use and self-regulation) decreased significantly (Z=2.11, 3.02, respectively, p < .05).

Table 1 shows relationships between participants’ test anxiety scores and their self-regulated learning scores. Participants’ test anxiety scores were positively associated with their cognitive strategy use scores in Year 1, but these became negatively associated in Year 2. Additionally, participants’ test anxiety and self-regulation scores became more negatively associated over time.

**Table 1:** Relationships between participants’ test anxiety and self-regulated learning scores

Test anxiety	Cognitive strategy use		Self-regulation	
	Pearson’s r	*p* value	Pearson’s r	*p* value
Year 1	0.33	(0.04)	-0.06	(0.73)
Year 2	-0.27	(0.87)	-0.35	(0.03)

## Discussion

Our findings show that medical students’ motivation and self-regulated learning change over time. Contrary to the literature suggesting students use more self-regulation strategies as they become more motivated,^2^^,^^13^ our study shows that even as medical students become more motivated, they actually use fewer self-regulated learning over time. These findings may be attributable to the lecture-driven curriculum that the study participants went through as the literature indicates pedagogy and the assessment system influence students’ motivation and self-regulated learning.^6^^, ^^14^ Still, our findings support the assertion made by White et al.^13^ that there is little evidence to suggest that medical schools are successfully helping medical students become effective self-regulated learners, in spite of such expectation held by many medical educators.

The most notable change in students’ MLSQ scores over time was in their test anxiety. This finding indicates students’ test anxiety increases by the end of Year 1 through their experiences with assessments in medical school. As research suggests that student’s test anxiety is negatively associated with his/her cognitive and metacognitive strategies use,^2^ it can be speculated that the decrease in students’ cognitive strategy use and self-regulation found in the present study is associated with their increased test anxiety. Our findings highlight the need for change in the medical school’s learning environment to lessen students’ test anxiety to facilitate their use of cognitive and meta-cognitive strategies.

The present study has a limitation in that it was performed with a small sample collected from a single site. Accordingly, future research is warranted with a larger sample to enhance the generalizability of our findings. Furthermore, our study investigated students over one year only when they were in the phase of the curriculum largely dependent on lectures. A longitudinal study is warranted to investigate whether the same pattern of changes continues when the learning environment shifts away from lectures and move towards a more self-directed one, such as problem-based learning courses and learning in clinical settings, as they progress through the years in medical school.

### 

The authors declare that they have no conflict of interest.

## References

[1] Zimmerman BJ (1990). Self-Regulated Learning and Academic Achievement: An Overview.. Educational Psychologist.

[2] Pintrich PR, de Groot EV (1990). Motivational and self-regulated learning components of classroom academic performance.. Journal of Educational Psychology.

[3] Stegers-Jager KM, Cohen-Schotanus J, Themmen AP (2012). Motivation, learning strategies, participation and medical school performance.. Med Educ.

[4] Brydges R, Butler D (2012). A reflective analysis of medical education research on self-regulation in learning and practice.. Med Educ.

[5] Wlodkowski RJ. Enhancing adult motivation to learn: a comprehensive guide for teaching all adults. 2nd Edition. San Francisco, CA: Jossey-Bass; 1998.

[6] Kusurkar RA, Ten Cate TJ, van Asperen M, Croiset G (2011). Motivation as an independent and a dependent variable in medical education: a review of the literature.. Med Teach.

[7] Pintrich PR, Schunk DH. Motivation in education: theory, research, and applications. 2nd Edition Englewood Cliffs, NJ: Prentice Hall; 2001.

[8] Zimmerman BJ, Martinez-Pons M (1990). Student differences in self-regulated learning: Relating grade, sex, and giftedness to self-efficacy and strategy use.. Journal of Educational Psychology.

[9] Barker JR, Olson JP. Medical students' learning strategies: evaluation of first year changes. J Miss Acad Sci. 1997; 42:96-100.

[10] Pizzimenti MA, Axelson RD (2015). Assessing student engagement and self-regulated learning in a medical gross anatomy course.. Anat Sci Educ.

[11] Cook DA, Thompson WG, Thomas KG (2011). The Motivated Strategies for Learning Questionnaire: score validity among medicine residents.. Med Educ.

[12] DeVellis RF. Scale development: theory and applications. Thousand Oaks, CA: Sage Publications; 2003.

[13] White CB, Gruppen LD, Fantone JC. Self-regulated learning in medical education. In: Swanwick T, editor. Understanding medical education: evidence, theory and practice. 2nd Edition. West Sussex, UK: Wiley Blackwell; 2014.

[14] White CB (2007). Smoothing out transitions: how pedagogy influences medical students' achievement of self-regulated learning goals.. Adv Health Sci Educ Theory Pract.

